# NiO/Perovskite Heterojunction Contact Engineering for Highly Efficient and Stable Perovskite Solar Cells

**DOI:** 10.1002/advs.201903044

**Published:** 2020-04-07

**Authors:** Bingjuan Zhang, Jie Su, Xing Guo, Long Zhou, Zhenhua Lin, Liping Feng, Jincheng Zhang, Jingjing Chang, Yue Hao

**Affiliations:** ^1^ State Key Discipline Laboratory of Wide Band Gap Semiconductor Tecchnology Shaanxi Joint Key Laboratory of Graphene Advanced Interdisciplinary Research Center for Flexible Electronics School of Microelectronics Xidian University 2 South Taibai Road Xi'an 710071 China; ^2^ Department of Applied Physical Sciences University of North Carolina at Chapel Hill Chapel Hill NC 27599 USA; ^3^ State Key Laboratory of Solidification Processing Northwestern Polytechnical University Xi'an Shaanxi 710072 P. R. China

**Keywords:** buffer layers, contact engineering, lattice mismatches, NiO, perovskite solar cells

## Abstract

Recent research shows that the interface state in perovskite solar cells is the main factor which affects the stability and performance of the device, and interface engineering including strain engineering is an effective method to solve this issue. In this work, a CsBr buffer layer is inserted between NiO*_x_* hole transport layer and perovskite layer to relieve the lattice mismatch induced interface stress and induce more ordered crystal growth. The experimental and theoretical results show that the addition of the CsBr buffer layer optimizes the interface between the perovskite absorber layer and the NiO*_x_* hole transport layer, reduces interface defects and traps, and enhances the hole extraction/transfer. The experimental results show that the power conversion efficiency of optimal device reaches up to 19.7% which is significantly higher than the efficiency of the device without the CsBr buffer layer. Meanwhile, the device stability is also improved. This work provides a deep understanding of the NiO*_x_*/perovskite interface and provides a new strategy for interface optimization.

## Graphical abstract

A CsBr buffer layer is inserted between NiO*_x_* hole transport layer and perovskite layer to relieve the lattice mismatch induced interface stress and induce more ordered crystal growth. The results show that the addition of the CsBr buffer layer optimizes the interface between the perovskite and NiO*_x_*, reduces interface defects and traps, and enhances the hole extraction/transfer.

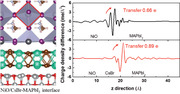

## 1 Introduction

Perovskite solar cells (PSCs) have attracted much attention for their rapid development in the power conversion efficiency (PCE) increase. From 2009 to 2019, the PCE has increased from 3.8% to 25.2%.^[^ ^1^, ^2^ ^]^ In addition, PSCs offer the advantages of simple fabrication, low cost, and the ability to produce transparent, flexible, and laminated devices.^[^ ^3^, ^4^ ^]^ The structures of PSCs can be divided into mesoporous structures and planar structures, wherein the planar structures include planar n‐i‐p structure and planar p‐i‐n structure.^[^ ^5^, ^6^, ^7^ ^]^ Among them, the PSCs with planar p‐i‐n structures have received increasing attention of researchers because of their low hysteresis and simple preparation process.^[^ ^8^, ^9^ ^]^

In the inverted structure PSCs, the hole transport layer (HTL) has a dominant influence on the device performance, and many p‐type semiconductor materials such as CuO, Cu_2_O, CuSCN, graphene oxide, poly(3,4‐ethylenedioxythiophene):poly(styrenesulfonate), and poly(bis(4‐phenyl)(2,4,6‐trimethylphenyl)amine) have been investigated and applied as the hole transport materials (HTMs) in the inverted structure PSCs.^[^ ^10^, ^11^, ^12^, ^13^, ^14^ ^]^ Compared with organic HTMs, inorganic HTMs have higher hole transporting ability, better stability, and lower cost. Among these HTMs, NiO*_x_*, as a wide bandgap (3.6–4.0 eV) p‐type semiconductor material, has been successfully employed in PSCs with inverted structure due to its suitable charge carrier mobility and proper work function which can well match the energy level of perovskites by adjusting the concentration of O^2−^ or Ni^2+^.^[^ ^15^, ^16^, ^17^, ^18^ ^]^

However, pristine NiO*_x_* has a high specific resistance of 10^8^ Ω^[^ ^19^ ^]^ and its low conductivity can aggravate the charge carrier recombination and degrade the hole extraction.^[^ ^20^ ^]^ One strategy to increase the NiO*_x_* conductivity is the doping method.^[^ ^15^, ^21^, ^22^, ^23^ ^]^ The nickel vacancies are considered as the main reason which dominates the p‐type conductivity in undoped NiO*_x_*.^[^ ^24^ ^]^ Since this kind of internal p‐type conductivity is limited because the Ni vacancies have large ionization energy in undoped NiO*_x_*, extrinsic dopants with shallower acceptor levels are commonly used to increase the conductivity.^[^ ^25^ ^]^ Previous reports have shown that doping Li,^[^ ^26^ ^]^ Co,^[^ ^27^ ^]^ Mg,^[^ ^28^ ^]^ or Cs^[^ ^22^ ^]^ in NiO*_x_* can significantly improve the electrical conductivity of NiO*_x_*, resulting in the enhanced device PCE.

In addition to the HTL deficiency, the interface defects between the perovskite layer and the NiO*_x_* layer seriously affect the charge carrier transfer since charge extraction only occurs at the interfaces, which may be particularly subject to charge recombination.^[^ ^29^ ^]^ Therefore, adding an interface layer between perovskite layer and the NiO*_x_* layer to reduce lattice mismatch induced interface defects and improve the matching of energy levels is a proper way to further improve the device performance of PSCs. Li and co‐workers inserted CsCl between TiO_2_ and perovskite in PSCs and this approach improves the device stability under UV exposure.^[^ ^30^ ^]^ Bai et al. applied a small molecule material diethanolamine to modify the NiO*_x_* nanocrystal film surface. The result showed that the conversion of PbI_2_ to MAPbI_3_ became slower, and better interface contact and improved film quality were obtained. Finally, highly efficient and stable devices with free hysteresis were achieved.^[^ ^31^ ^]^ However, the detailed mechanism of using alkali metal halide buffer layer to reduce the lattice mismatch and improve interface contact quality is still not clear, and the use of CsBr as a buffer layer to modulate the crystal contact properties between HTL/perovskite has rarely been investigated.

Herein, we applied CsBr as the buffer layer between the NiO*_x_* layer and the perovskite layer to modulate the contact properties. It was found that the CsBr buffer layer could relieve the interface stress, enhance the perovskite film quality with large grain size, increase charge extraction efficiency, and reduce the charge recombination. The detailed mechanism is analyzed by various techniques and first‐principle calculations. Finally, PSCs with high PCE of 19.7% and good device stability were achieved.

## 2 Results and Discussions

**Figure 1:**
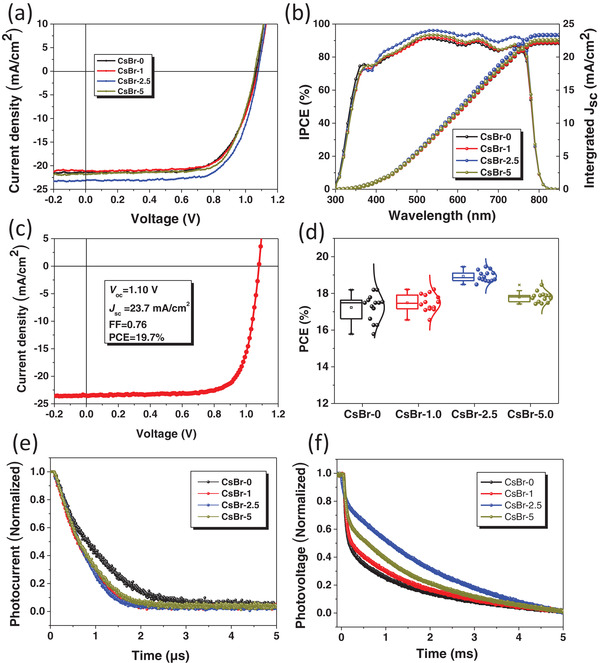
a) *J*–*V* curves of perovskite solar cells based on different concentrations of CsBr. b) The IPCE spectra for the device based on different concentrations of CsBr. c) The best *J*–*V* characteristic for device based on 2.5 mg mL^−1^ CsBr. d) Device parameters of the perovskite devices based on different concentrations of CsBr. e) Transient photocurrent and f) transient photovoltage measurements of solar cells based on different concentrations of CsBr.

PSC devices with configuration of indium tin oxide (ITO)/NiO*_x_*/CsBr/MA_1−_ *_x_*FA*_x_*PbI_3−_ *_y_*Cl*_y_*/phenyl‐C61‐butyric acid methylester/bathocuproine/Ag were first fabricated to study the effects of CsBr buffer layer on device performance. The architecture of the device with CsBr buffer layer is presented in Figure S1 in the Supporting Information, where the electron transport layer (ETL) and HTL utilized are phenyl‐C61‐butyric acid methyl ester (PC_61_BM) and NiO*_x_*, respectively. The whole device fabrication process with more details is presented in the experimental part. CsBr modified PSCs were prepared by inserting CsBr layer with different concentrations. The applied CsBr concentrations were 0, 1, 2.5, and 5 mg mL^−1^ and the resulted films were denoted as CsBr‐0, CsBr‐1, CsBr‐2.5, and CsBr‐5, respectively. The current density–voltage (*J*–*V*) curves of devices based on CsBr buffer layer treatment with different concentrations are exhibited in **Figure** **1** and the extracted corresponding device photovoltaic parameters are summarized in **Table** **1**. As shown in Figure 1 and Table 1, it can be observed that the 2.5 mg mL^−1^ CsBr modified devices achieve the best performance with a short circuit current density (*J*_sc_) of 23.5 mA cm^−2^, an open circuit voltage (*V*_oc_) of 1.09 V, a fill factor (FF) of 0.75, and an average PCE of 19.2%, as measured via current voltage measurements under simulated AM1.5 sun light at 100 mW cm^−2^ irradiance. This PCE is significantly improved compared to the control device (average PCE: 17.7%, *J*_sc_: 22.7 mA cm^−2^, *V*_oc_: 1.07 V, and FF: 0.74). *V*_oc_, FF, and *J*_sc_ are obviously improved when CsBr is applied, leading to a substantially improved average PCE, as shown in Figure 1 and Table 1.

**Table 1:** Average device parameters of perovskite solar cells based on different concentrations of CsBr. The average results are derived from 20 perovskite solar cells

	*J*_sc_ [mA cm^−2^]	*V*_oc_ [V]	FF [%]	PCE [%]	*R*_s_ [Ω cm^2^]	*R*_sh_ [kΩ cm^2^]
CsBr‐0	22.7	1.07	73.7	17.7	7.81	263.05
CsBr‐1	22.7	1.09	74.1	18.3	6.66	142.34
CsBr‐2.5	23.5	1.09	75.1	19.2	4.87	413.34
CsBr‐5	23.1	1.08	74.9	18.7	6.52	294.05

For the *J*_sc_ enhancement of the CsBr modified device, the possible main causes are the improved charge transfer and the better film crystallinity and they will be discussed in detail later. Incident photon‐to‐current conversion efficiency (IPCE) spectra (Figure 1b) were also measured to check the device *J*_sc_, and it can be seen that the integrated *J*_sc_ values (CsBr‐0: 21.08 mA cm^−2^, CsBr‐1: 21.11 mA cm^−2^, CsBr‐2.5: 22.08 mA cm^−2^, and CsBr‐5: 21.49 mA cm^−2^) obtained from the IPCE spectra are well consistent with those extracted from the measured *J*–*V* curves (CsBr‐0: 22.7 mA cm^−2^, CsBr‐1: 22.7 mA cm^−2^, CsBr‐2.5: 23.5 mA cm^−2^, and CsBr‐5: 23.1 mA cm^−2^). High IPCE values not only mean the efficient photon to electricity conversion but also suggest less charge carrier recombination at the interface between NiO*_x_* film and pervoskite. Figure 1d shows the device statistic results, and it can be seen that the CsBr modified device exhibits narrow parameter distribution. The reason for the increase in *J*_sc_ and FF is attributed to the fast charge transfer and the reduction of interface defects related recombination. Moreover, the reason for the *V*_oc_ increase is attributed to the better energy level alignment as revealed by ultraviolet photoelectron spectroscopy (UPS) spectra later. In addition, the data in Figure 1d indicate a good reliability of the conditions. The device series resistance (*R*_s_) and shunt resistance (*R*_sh_) are evaluated to deeply understand the performance improvement. Compared to the control device (*R*_s_: 7.81 Ω cm^2^, *R*_sh_: 263.05 kΩ cm^2^), the devices with CsBr‐modification have decreased *R*_s_ (4.87 Ω cm^2^) and enlarged *R*_sh_ (413.34 kΩ cm^2^), suggesting improved charge transfer and reduced charge recombination due to the CsBr modification, which agrees with the device photovoltaic performance.

The device transient photocurrent (TPC) and photovoltage (TPV) measurements were carried out to further verify these results. TPC measurement is usually used to characterize the charge extraction and transfer process in the solar cell devices, and the charge‐extraction lifetime can be obtained from TPC curves. Generally, the reduced charge transfer lifetime also indicates reduced trap density.^[^ ^12^ ^]^ As shown in Figure 1e, the photocurrent decay of CsBr‐*x* (*x* = 0, 1, 2.5, and 5) modified devices is measured under short‐circuit condition. Faster decay (CsBr‐1: 0.68 µs, CsBr‐2.5: 0.64 µs, and CsBr‐5: 0.70 µs) is found for the CsBr modified device than that for the control device (CsBr‐0: 0.98 µs), which means that much more efficient charge extraction and transfer occur in the CsBr‐modified device, resulting in improved *J*_sc_ and FF. The charge‐recombination lifetime can be determined for TPV measurement (Figure 1f). Under open‐circuit condition, CsBr‐modified devices exhibit longer charge recombination lifetimes (CsBr‐1: 1.73 ms, CsBr‐2.5: 2.81 ms, and CsBr‐5: 1.79 ms) compared to the control device (CsBr‐0: 1.55 ms), indicating the efficiently suppressed charge recombination and reduced trap density for the CsBr‐modified devices. The photoluminescence (PL) measurements also show consistent results that CsBr‐modified devices exhibit improved charge transfer and decreased trap states.

**Figure 2:**
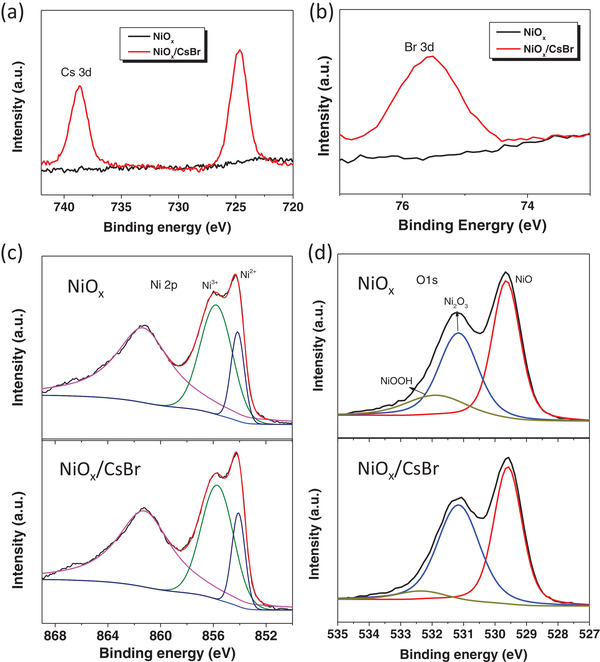
XPS spectra of the a) Cs 3d, b) Br 2p, c) Ni 2P, and d) O 1s of NiO*_x_* films without and with CsBr modifications.

In the following part, the 2.5 mg mL^−1^ CsBr treatment concentration is selected, since this CsBr treatment gives the best performance, and the corresponding condition is labeled as NiO*_x_*/CsBr. The effect of CsBr buffer layer on NiO*_x_* was investigated first. X‐ray photoelectron spectroscopy (XPS) measurements were taken to do the analysis of the element compositions of CsBr modified NiO*_x_* and pristine NiO*_x_* thin films. The XPS spectra of Cs 3d and Br 3d are shown in **Figure** **2**a,b, respectively, to verify the existence of CsBr. This situation indicates that CsBr has been successfully deposited on the NiO*_x_* surface. Figure 2c,d shows the XPS spectra of the Ni 2p and O 1s with the typical peaks corresponding to Ni^2+^ (853.8 eV) and Ni^3+^ (855.6 eV) observed in the films.^[^ ^32^, ^33^ ^]^ It can be seen that the CsBr modification has no effect on the composition of NiO*_x_* film. From Figure 2c,d, it is found that the binding energy of modified NiO*_x_* decreases 0.1 eV compared with the unmodified NiO*_x_* at 854.5 eV, indicating that CsBr affects the distribution of electrons in Ni. While the Ni^3+^/Ni^2+^ ratio does not change and keeps at 9.4. It can also be seen from Figure 2d that with the CsBr modification, the peak intensity at 532 eV slightly decreases, indicating that the CsBr modification can reduce the content of NiOOH.

**Figure 3:**
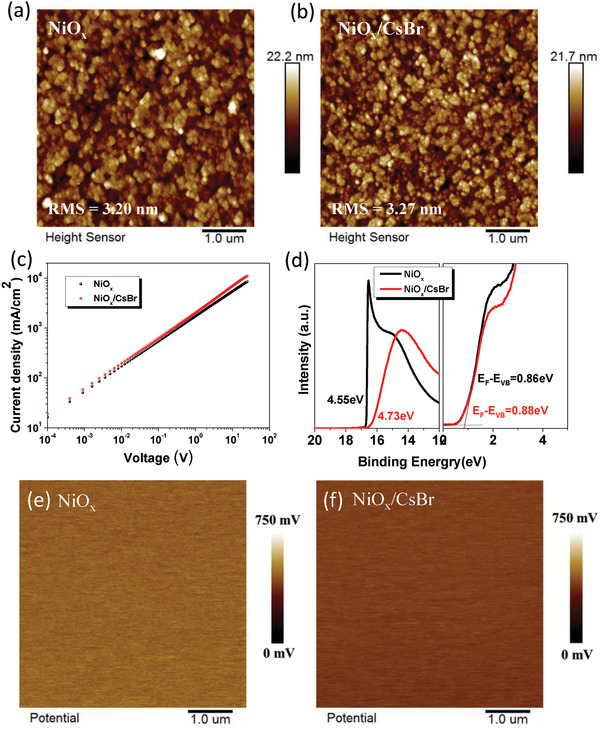
AFM images of a) NiO*_x_* and b) NiO*_x_*/CsBr films. c) The space‐charge‐limited current (SCLC) curves of NiO*_x_* films without and with CsBr treatment with a structure of ITO/NiO*_x_*/Ag. d) UPS spectra of the work function region and valence band region for NiO*_x_* film and NiO*_x_*/CsBr film. e,f) KPFM images of NiO*_x_* films without and with CsBr treatment.

The surface morphology of NiO*_x_* treated without and with CsBr was studied by atomic force microscopy (AFM) measurement. As shown in **Figure** **3**a,b, the root mean square values under different conditions are 3.20 nm (NiO*_x_*), and 3.27 nm (NiO*_x_*/CsBr), respectively. Hence, the CsBr modification has less effect on the surface morphology of NiO*_x_* films. Since the device performance can be improved by optimizing carrier mobility of ETL, current–voltage characteristics of the single‐carrier devices were measured and the charge‐carrier mobility can be calculated.^[^ ^34^ ^]^ Figure 3c shows the current versus voltage on a logarithmic scale for the single‐carrier device. The hole‐only device architecture is ITO/NiO*_x_*/CsBr/Ag. According to the previous reports, the curves can be divided into three regions, linear ohmic regime, trap‐filled regime, and space charge limited current (SCLC) regime.^[^ ^35^ ^]^ Under this condition, a Mott–Gurney Law can be applied for the SCLC transport within the single‐carrier devices.^[^ ^36^, ^37^ ^]^ Based on the Mott–Gurney law, the estimated mobility values ​​are 9.94 × 10^−3^ and 1.31 × 10^−2^ cm^2^ V^−1^ s^−1^ for NiO*_x_* and NiO*_x_*/CsBr, respectively. The above results show that the mobility of CsBr modified NiO*_x_* film has been improved, probably because Cs^+^ is slightly doped into NiO*_x_*, which improves the conductivity and mobility.

The energy levels of NiO*_x_* and NiO*_x_*/CsBr films were measured with UPS, as shown in Figure 3d. The valence band maximum (VBM) values can be obtained according to the formula of VBM = *W*_F_ + (*E*_F_ − *E*_VB_), where *W*_F_ is work function, *E*_F_ is Fermi level, and *E*_VB_ is valence band energy level.^[^ ^38^ ^]^ The VBM energies of NiO*_x_* and NiO*_x_*/CsBr film are calculated to be 5.41 and 5.84 eV, respectively. The work function of the combustion NiO*_x_* and the perovskite valence band (5.9 eV) are well aligned, which is beneficial for hole transporting and electron blocking. Kelvin probe force microscopy (KPFM) was further used to investigate the CsBr effect on the electronic energy levels of NiO*_x_* films. As shown in Figure 3e,f, the surface potential of NiO*_x_* film with CsBr treatment is smaller than that of pristine NiO*_x_* film, indicating that the CsBr treatment slightly increases the work function of NiO*_x_* (Δ*W*_F_ = 0.064 eV). This is consistent with UPS results.

**Figure 4:**
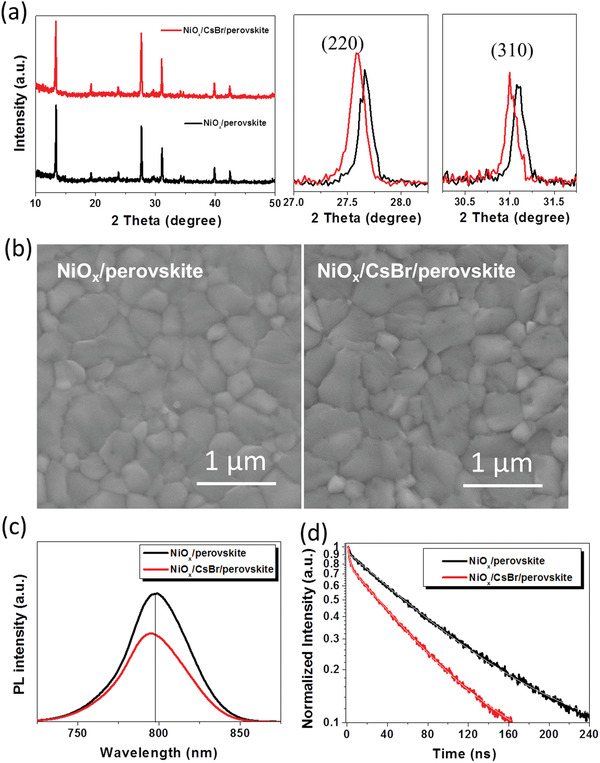
a) XRD patterns and the zoom‐in spectra of (220) and (310) peaks of the perovskite thin films modified without and with CsBr. b) SEM images of the perovskite thin films modified without and with CsBr. c) PL and d) TR‐PL spectra of perovskite thin films modified without and with CsBr buffer layer excited from NiO*_x_* side.

Besides the effects on NiO*_x_* films, we have investigated the effects of the CsBr buffer on perovskite films. By considering the thermodynamic properties mismatch between the NiO*_x_* and perovskite,^[^ ^39^, ^40^, ^41^, ^42^ ^]^ the large thermal expansion coefficient (4.0 × 10^−5^ K^−1^) and low Young's modulus (17.8 GPa) of perovskite can make the perovskite undergo significant lattice strain when the perovskite films are thermally annealed on the hot plate at 100 °C, as reported by the previous studies.^[^ ^43^, ^44^, ^45^ ^]^ While the thermal expansion coefficient (4.2 × 10^−5^ K^−1^)^[^ ^46^ ^]^ and Young's modulus (16.2 GPa)^[^ ^47^ ^]^ of CsBr are close to those of perovskite, which indicates that the small deviation between CsBr and perovskite can release the lattice strain of perovskite during the process of perovskite film formation. To further verify such phenomenon, **Figure** **4**a displays the evolution of X‐ray diffraction (XRD) patterns of the perovskite thin films modified with CsBr. Both two films exhibit three main diffraction peaks, which is corresponding to the (110), (220), and (310) diffraction peaks, respectively. It can be seen from Figure 4a that the peak intensity of the perovskite pattern modified with CsBr slightly increases (zoom‐in spectra shown in Figure 4a and Figure S2, Supporting Information). Moreover, the CsBr has an obvious effect on the peak position at (110), (220), and (310). When the CsBr buffer layer is introduced, the peak position shifts to lower degree, indicating that the lattice strain of perovskite has been relaxed, which is similar with previously reported results.^[^ ^48^ ^]^ Meanwhile, this indicates that the CsBr mainly acts as an interlayer since the XRD peaks should shift to larger angles, if the larger MA cation or I anion were substituted by Cs cation or Br anion. This is consistent with previously reported results^[^ ^17^ ^]^ and also confirmed by our element analysis by energy dispersive X‐ray spectroscopy results (Table S1, Supporting Information). It was thought that the CsBr buffer layer can induce a more ordered crystal structure and crystal orientation. Such normalized structures of perovskites by CsBr buffer layers can be further intuitively observed from the optimized structures of NiO/MAPbI_3_ interfaces with and without CsBr buffer layer in Figure S3 in the Supporting Information. It is evidently that the distortion of PbI_6_ octahedron at the interface is significantly normalized upon introducing CsBr buffer layer into the NiO/MAPbI_3_ interface since the lattice constants (12.81 Å) of CsBr (110) surface more closely match those of MAPbI_3_ (110) surface (12.85 Å) compared to those of NiO (100) surface (12.45 Å). At the same time, the thermal expansion coefficient (4.20 × 10^−5^ K^−1^) of CsBr is closer to that of MAPbI_3_ (≈4.0 × 10^−5^ K^−1^) compared to that of NiO (1.38 × 10^−5^ K^−1^), leading to the weaker external stress for PbI_6_ octahedron. Thus, the thin CsBr acts as a template for perovskite formation and reduces the crystal lattice mismatch, which is similar with the 2D materials induced crystal growth.^[^ ^49^ ^]^ Moreover, the narrowed the full width half maximum of perovskite thin film based on NiO*_x_*/CsBr indicates that perovskite thin film possesses an optimal crystalline feature with less crystal imperfections and structure defects (Figure S2, Supporting Information).

Figure 4b exhibits the scanning electron microscopy (SEM) images of perovskite films with and without CsBr modification. It is found that the average crystal size of perovskite film increases from 400 to 440 nm from pristine NiO*_x_* to NiO*_x_*/CsBr film, which is calculated by using Nano Measurer software (Figure S4, Supporting Information). The larger perovskite grains indicate that the surface of the NiO*_x_* modified with CsBr is more conducive to the crystallization of perovskite film. Steady‐state PL and time‐resolved PL (TR‐PL) spectroscopy measurements were used to investigate the film quality and charge transfer kinetics between perovskite and NiO*_x_*. Since the laser penetration depths under the excitation wavelength of 510 nm are smaller than the perovskite layer thickness used in this study (≈630 nm), the PL behavior is excitation dependent. When the PL measurement is excited from the NiO*_x_* side, there is a blueshift from 797 nm (NiO*_x_*) to 795 nm (NiO*_x_*/CsBr) in the PL peak shows in Figure 4c, suggesting effectively passivated trap states due to the improved quality and crystallinity of the perovskite film. In addition, a significant decrease can be clearly observed in PL intensity and PL lifetime for CsBr‐modified device. The steady‐state PL is quenched more strongly when the perovskite film is formed on NiO*_x_*/CsBr. Similar results are revealed in the TR‐PL spectra (see in Figure 4d). The carrier lifetime can be calculated by the PL curve fitting with a double exponential decay model.^[^ ^50^ ^]^ It is found that the average decay lifetime (τ_ave_) of the perovskite film modified with CsBr (59.8 ns) is much shorter than that of the perovskite film without CsBr modification (83.5 ns). These results indicate that faster electron extraction occurs at the CsBr‐modified NiO*_x_*/perovskite interface compared to the interface without CsBr modification. When the PL measurements are excited from air side, in this case, the PL intensity is determined by the radiative recombination of the top perovskite film. And the PL intensity and lifetime are mainly dependent on the perovskite film quality.^[^ ^16^ ^]^ As shown in Figure S5 in the Supporting Information, the PL intensity and lifetime increase (from 424 to 618 ns) for NiO*_x_*/CsBr based perovskite film compared to NiO*_x_* based perovskite film.

**Figure 5:**
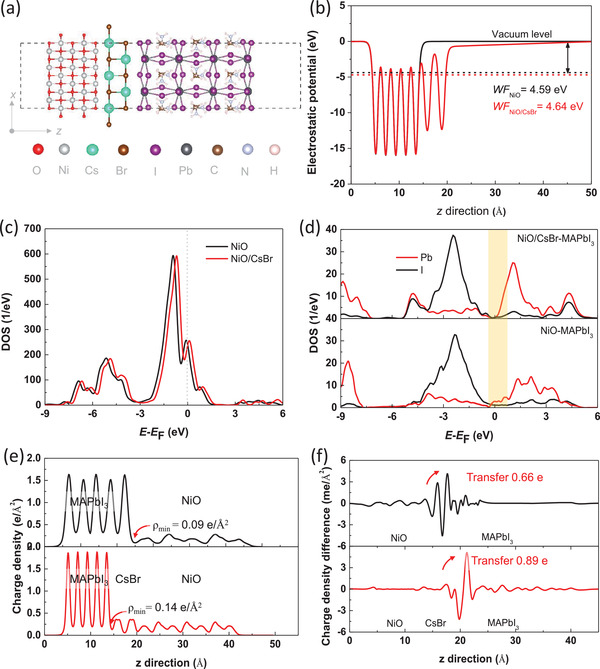
a) Side view of NiO/CsBr‐MAPbI_3_ interface. b) Electrostatic potential and c) density of states of isolated NiO and NiO/CsBr surface. d) Density of states of MAPbI_3_ surface in NiO/MAPbI_3_ and NiO/CsBr‐MAPbI_3_ interfaces. e) Charge density and f) charge density difference coupling with bader charge of NiO/MAPbI_3_ and NiO/CsBr‐MAPbI_3_ interfaces.

To obtain more information on how the CsBr buffer layer affects the performance of NiO/perovskite interface, first‐principle calculations of NiO/CsBr, CsBr/MAPbI_3_, NiO/MAPbI_3_, and NiO/CsBr‐MAPbI_3_ interfaces were performed. The NiO/CsBr‐MAPbI_3_ interfaces are designed by 3 × 3 NiO (100) surfaces, 3 × 2 CsBr (110) surfaces, and 1 × 1 MAPbI_3_ (110) surfaces, as displayed in **Figure** **5**a. The strains of MAPbI_3_ (110) surfaces in NiO/MAPbI_3_ and NiO/CsBr‐MAPbI_3_ interfaces are less than 7% and 5%, respectively. Without a CsBr buffer layer, NiO surface with a large work function of about 4.59 eV shows obvious p‐type conductivity. Upon combining with the CsBr buffer layer, as displayed in Figure 5b,c, the Fermi level shifts toward the lower energy and far away to the VBM, resulting in a larger work function and stronger p‐type conductivity for NiO*_x_* surface, which is consistent with above experimental results. The enhanced work function can increase the valence band offset between NiO and MAPbI_3_ surface and then lead to a larger activation energy for carrier recombination.^[^ ^51^, ^52^ ^]^ Meanwhile, the interfacial gap states (as the density of states around the Fermi level and marked in the Figure 5d) of NiO/CsBr‐MAPbI_3_ interface are smaller than those of NiO/MAPbI_3_ interface. According to the previous study, such weakened interfacial gap states suggest the reduced carrier recombination at the interface region, which is beneficial to improve the fill factor of PSC. Thus, upon introducing a CsBr buffer layer into NiO/MAPbI_3_ interface, the fill factor of PSC is enhanced in experiment. In addition, Figure 5b demonstrates the average charge density in the *x*–*y* planes normal to the *z*‐axis. High charge density at the interface indicates low contact resistance and allows sufficient injection of charge into the perovskite surface.^[^ ^53^ ^]^ The minimum charge density (ρ_min_) at the NiO/CsBr‐MAPbI_3_ interface of about 0.14 e Å^−2^ is higher than that at the NiO/MAPbI_3_ interface of about 0.09 e Å^−2^. It suggests that the CsBr buffer layer can improve the resistance of NiO/MAPbI_3_ interface and the current density at interface and then enlarge the *J*_sc_ of PSC. To further confirm such phenomenon, the charge density difference and bader charge transfer at the NiO/CsBr‐MAPbI_3_ and NiO/MAPbI_3_ interfaces are further given in Figure 5f. Obviously, when a CsBr buffer layer is added into the NiO/MAPbI_3_ interface, the transferred electron into perovskite surface from about 0.66 to 0.89 e. Therefore, the *J*_sc_ of PSC is evidently enlarged when a CsBr buffer layer is introduced between NiO HTL and perovskite layer.

**Figure 6:**
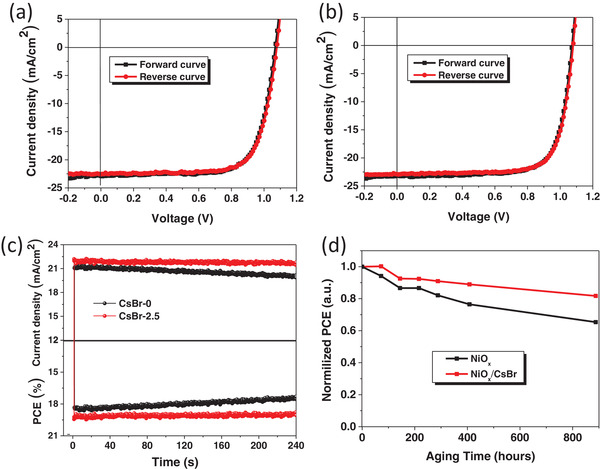
a,b) *J*–*V* curves of perovskite solar cells without and with CsBr buffer layer measured under different scan directions. c) Steady output characteristics of devices without and with CsBr buffer layer measured at maximum power point. d) The air stability of unencapsulated devices based on NiO*_x_* hole transporting layers without and with CsBr buffer layer.

The hysteresis issue which may cause the device performance underestimation or overestimation always cannot be avoided in PSC devices. The hysteresis behavior is a complex process since many factors like crystal defects, perovskite grain boundaries, and interface defects, ferroelectricity, ion migration, etc. can affect it.^[^ ^54^, ^55^ ^]^ The hysteresis behaviors of PSCs with CsBr as the buffer layer are also studied. **Figure** **6** and Table S1 in the Supporting Information show the cell measured *J*–*V* curves along the backward scan direction (1.2–−0.2 V) and forward scan direction (−0.2–1.2 V). Figure 6a,b shows that the hysteresis behavior of CsBr‐modified device is negligible and similar as that of the pristine NiO*_x_* based device, suggesting the efficient hole extraction ability of CsBr‐modified perovskite thin film due to the good film quality. The device performance reliability was investigated by measuring the characteristics of the steady‐state output of current density and PCE values at maximum power point. The steady‐state PCEs as shown in Figure 6c are measured to be 18.0% (NiO*_x_* based device), and 19.2% (NiO*_x_*/CsBr base device), and the steady‐state current densities are 20.6 mA cm^−2^ (NiO*_x_* based device) and 21.8 mA cm^−2^ (NiO*_x_*/CsBr based device), which is consistent with the values extracted from measured *J*–*V* curves, indicating the measurement reliability. It is worth noticing that this performance of pristine devices has a performance decline over the longer time, while the modified device has a stable performance output due to the improved interface contact.

Finally, the device stability was investigated by storing the device in the ambient air with RH% ≈30% for 900 h. As shown in Figure 6d, the PCE of control device declines to 65% of initial efficiency after 900 h while the device modified by CsBr still maintains 82% of the initial efficiency during the same period. This indicates that the air stability of the devices with CsBr buffer layer is enhanced, since the CsBr buffer layer can improve the perovskite/HTL interface contact and the perovskite film quality.^[^ ^30^, ^31^ ^]^ Generally, the device stability is much dependent on the perovskite/HTL interface which could cause water invasion or penetration into interface between perovskites and NiO*_x_*. The improved interface contact can significantly reduce the interface defects induced degradation and enhance the device stability.^[^ ^56^ ^]^

## 3 Conclusion

In conclusion, this study reports the method of optimizing the hole transport layer and the perovskite absorber layer heterojunction contact (NiO*_x_*/Perovskite) with CsBr buffer layer. By applying this modification method in planar heterojunction PSCs with modified two‐step method fabricated perovskite film, the device efficiency is improved to 19.7%. The enhanced performance of the device is attributed to relieved lattice stress, better film quality, enhanced hole mobility, improved charge extraction/transfer, and reduced interface traps/defects related recombination. The PSC device also exhibits good environmental stability. This interface modification method is simple and scalable. It is compatible with the low cost flexible energy devices, and can facilitate the future commercialization of perovskite‐based photovoltaic technology.

## 4 Simulation Section

The first‐principle calculations are performed in the framework of density functional theory (DFT) using the Vienna Ab Initio Simulation Package code. The Perdew–Burke–Ernzerhof functional with the projector augmented wave method is used for geometry optimization and self‐consistent calculations. The van der Waals interaction is considered using DFT‐D2 method. The cutoff energy of 400 eV and Monkhorst–Pack *k*‐point meshes spanning less than 0.015 Å^−3^ in the Brillouin zone are chosen. The convergence criteria for energy change and maximum force are set as 10^−5^ eV and 0.01 eV Å^−1^, respectively. Vacuums of 20 Å are added perpendicular to the interfaces to minimize the artificial interlayer interactions.

## Conflict of Interest

The authors declare no conflict of interest.

## Supporting information

Supporting Information
