# Supplementary material for: NiO/Perovskite Heterojunction Contact Engineering for Highly Efficient and Stable Perovskite Solar Cells
Source: Adv Sci (Weinh). 2020 Apr 7;7(11):1903044. doi: 10.1002/advs.201903044 (PMC7284208; doi:10.1002/advs.201903044)
Supplement: Supplementary file 1 — Supporting Information [file ADVS-7-1903044-s001.pdf]

Copyright WILEY-VCH Verlag GmbH & Co. KGaA, 69469 Weinheim, Germany, 2020.

ADVANCED SCIENCE Open Access

Supporting Information

for *Adv. Sci.,* DOI: 10.1002/advs.201903044

NiO/Perovskite Heterojunction Contact Engineering for Highly Efficient and Stable Perovskite Solar Cells

Bingjuan Zhang, Jie Su, Xing Guo, Long Zhou, Zhenhua Lin, Liping Feng, Jincheng Zhang, Jingjing Chang,* and Yue Hao*

((Supporting Information can be included here using this template))

Copyright WILEY-VCH Verlag GmbH & Co. KGaA, 69469 Weinheim, Germany, 2020.

Supporting Information

NiO/Perovskite Heterojunction Contact Engineering for Highly Efficient and Stable Perovskite Solar Cells

Bingjuan Zhang,[a] Jie Su,[a,b] Xing Guo,[a] Long Zhou,[a] Zhenhua Lin,[a] Liping Feng,[c] Jincheng Zhang,[a,b] Jingjing Chang,[a,b]* Yue Hao[a,b]*

B, Zhang, Dr. J. Su, X. Guo, L. Zhou, Dr. Z. Lin, Prof. J. Zhang, Prof. J. Chang, Prof. Y. Hao
State Key Discipline Laboratory of Wide Band Gap Semiconductor Tecchnology, Shaanxi Joint Key Laboratory of Graphene, Advanced Interdisciplinary Research Center for Flexible Electronics, School of Microelectronics, Xidian University, 2 South Taibai Road, Xi'an, 710071, China. Email: zhlin@xidian.edu.cn, jjingchang@xidian.edu.cn
Prof. L. Feng,
State Key Laboratory of Solidification Processing, Northwestern Polytechnical University, Xi'an, Shaanxi, 710072, P. R. China.

Experimental Section

Materials: All the materials, such as nickel nitrate ($Ni(NO_3)_2 \cdot 6H_2O$), cesium bromide (CsBr), MAI (99.9 %, Dyesol), FAI (99.9 %, Dyesol), lead (II) iodide (PbI_2, 99.999 %, Alfa), lead (II) chloride ($PbCl_2$, 99.999 %, Alfa), phenyl-C61-butyric acid methyl ester ($PC_{61}BM$, 98 %, nano-c), N,N′-dimethylformamide (99.8 %, Aladdin), chlorobenzenes (99.8 %, Sigma), isopropanol (99.5 %,Sigma), bathocuproine (96 %, Alfa), 2-methoxyethanol, acetylacetone(99.8 %, Sigma) and Methanol were used as received without further purification. The CsBr modification layer was prepared by dissolving CsBr in Methanol to form 1 mg/mL, 2.5 mg/mL and 5.0 mg/mL CsBr solution.

Film Formation and Device Fabrication: Planar PSCs were fabricated on prepatterned indium tin oxide (ITO) glass substrates (around 2 × 2.5 cm^2 in size, 10 Ω per square). The

patterned transparent conducting oxide (TCO) glass substrates were sequentially ultrasonic cleaned with detergent, deionized water, acetone, and IPA at 50 ^{o}C for 20 min, respectively. Then, the TCO substrates were dried with nitrogen and treated in a UV ozone oven for 15 min. The NiO_x hole transport layer was made by dissolving 270.79 mg $Ni(NO_3)_2 \cdot 6H_2O$ (1.0 mmol) in 2-methoxyethanol (10 mL). After the solution was stirred at 50 ^{o}C for1 h, 100 μL acetylacetone was added to the solution, and then the solution was further stirred overnight at room temperature. This method is based on the method reported in Liu's article.[1] A thin layer of NiO_x was spin coated on the substrates at 3000 rpm for 45 s and annealed at 250 ^{o}C for 45 min. Cool down the temperature of NiO_x-covered TCO substrates to room temperature. Then, a thin layer of CsBr was spin coated on the NiO_x-covered TCO substrates at 3000 rpm for 30 s and annealed at 250 ^{o}C for 10 min in air. After that, the substrates were transferred into a nitrogen-filled glove box. PbI_2 (1.36 mol) and $PbCl_2$ (0.24 mol) were dissolved in the solvent of DMF and stirred for 2 h at 75 ^{o}C. MAI (70 mg) and FAI (30 mg) were dissolved in the solvent of IPA with 0.9 vol% DMF. Around 60 μL PbX_2 precursors preheated to 75 ^{o}C were transferred by pipettes to the NiO_x-covered TCO substrates. The spin coating process was programmed to run at 3000 rpm for 45 s. Then, MAI was spin coated on top of the dried PbI_2 layer at room temperature at 3000 rpm for 45 s. All of the films were thermally annealed on the hot plate at 100 ^{o}C for 10 min. Next, a layer of PCBM (20 mg/mL in chlorobenzene) was spin coated on the top of the perovskite layer at 2000 rpm for 40 s. After that, a thin layer of BCP (0.5 mg/mL in IPA) was spin coated on the top of the PCBM layer. The devices were finished by thermally evaporated 100 nm Ag. All the devices had an effective area of 7.5 mm^2 defined by the metal mask.

Materials and Device Characterization: The morphology measurement of the perovskite layers was taken by SEM (JSM-7800F). XRD test was conducted on Bruker D8 Advance

XRD. XPS experiments were carried out on the Escalab 250Xi using monochromatic Al-Ka (1486.6 eV) as the radiation source. The film morphology was investigated by a tapping mode atomic force microscopy (AFM) (Bruker ICON-PKG AFM). Space charge limited current (SCLC) and photoluminescence spectra were collected on an Edinburgh Instruments FLS920, and the excitation wavelength was 633 nm. The UV–vis absorption spectra were recorded with an UV–visible spectrophotometer (Perkin-Elmer Lambda 950). Photovoltaic performances were measured by using a Keithley 2400 source meter under simulated sunlight from XES-70S1 solar simulator matching the AM 1.5G standard with an intensity of 100 mW/cm^2. The system was calibrated against an NREL certified silicon reference solar cell. IPCEs of PSCs were measured by the solar cell quantum efficiency measurement system (SCS10-X150, Zolix instrument. Co. Ltd). TR-PL spectra were measured using the Pico Quant Fluotime 300 by using a 510 nm picosecond pulsed laser. Transient photocurrent measurement was performed with a system excited by a 532 nm (1000 Hz, 3.2 ns) pulse laser. Transient photovoltage measurement was performed with the same system excited by a 405 nm (50 Hz, 20 ms) pulse laser. A digital oscilloscope (Tektronix, D4105) was used to record the photocurrent or photovoltage decay process with a sampling resistor of 50 Ω or 1 MΩ, respectively.

First-principles calculations: The first-principle calculations were performed in the framework of density functional theory (DFT) using the VASP code. The Perdew-Burke-Ernzerhof (PBE) functional with the projector augmented wave (PAW) method was used for geometry optimization and self-consistent calculations. The van der Waals interaction was considered using DFT-D2 method. The cut-off energy of 400 eV and Monkhorst-Pack k-point meshes spanning less than 0.015 $Å^{-3}$ in the Brillouin zone were chosen. The convergence criteria for energy change and maximum force were set as 10^{-5} eV and 0.01 eV/Å,

respectively. Vacuums of 20 Å were added perpendicular to the interfaces to minimize the artificial interlayer interactions.

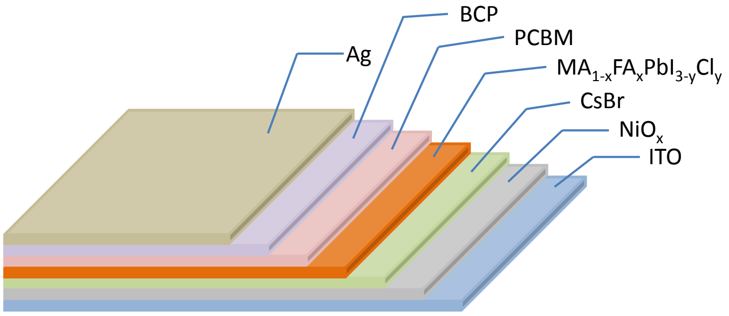

Figure S1. The device architecture of the inverted planar PSCs

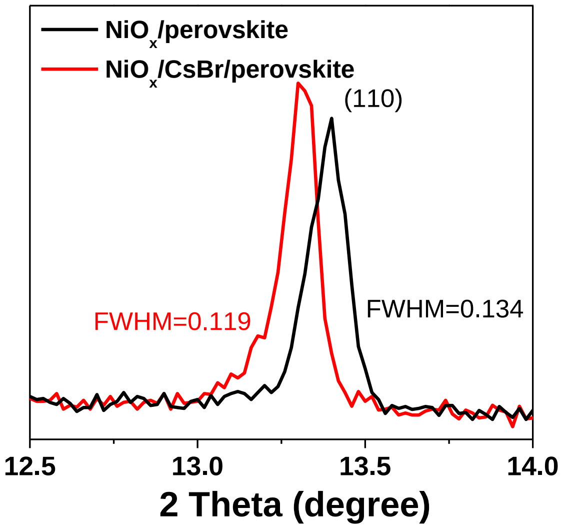

Figure S2. XRD spectra of perovskite films deposited on pristine NiO_x and CsBr modified NiO_x thin films.

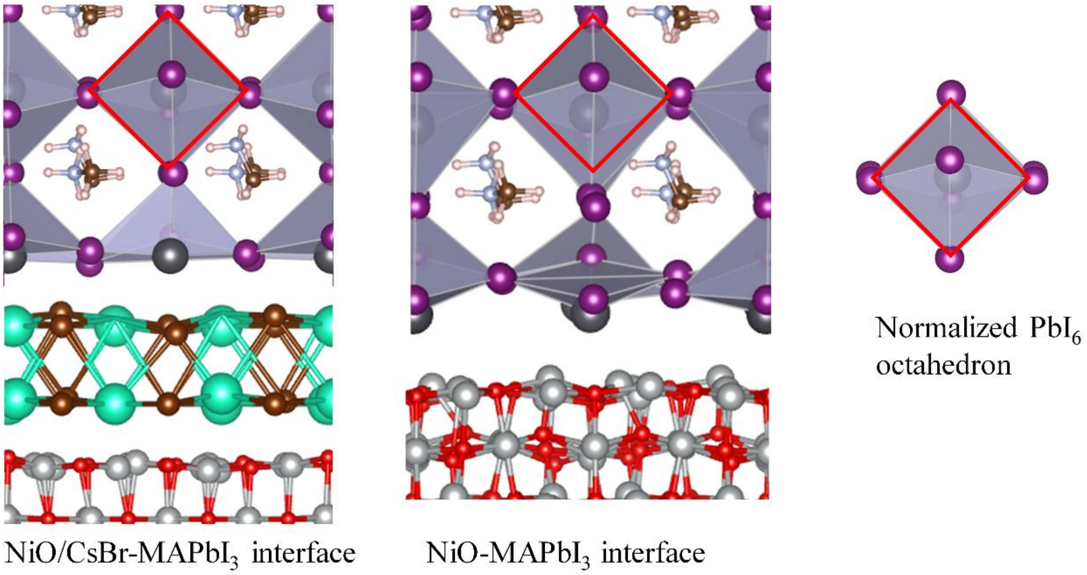

Figure S3. Optimized structures of NiO-$MAPbI_3$ interface with and without CsBr interfacial layer. The normalized PbI_6 octahedron of $MAPbI_3$ is presented by the red frame.

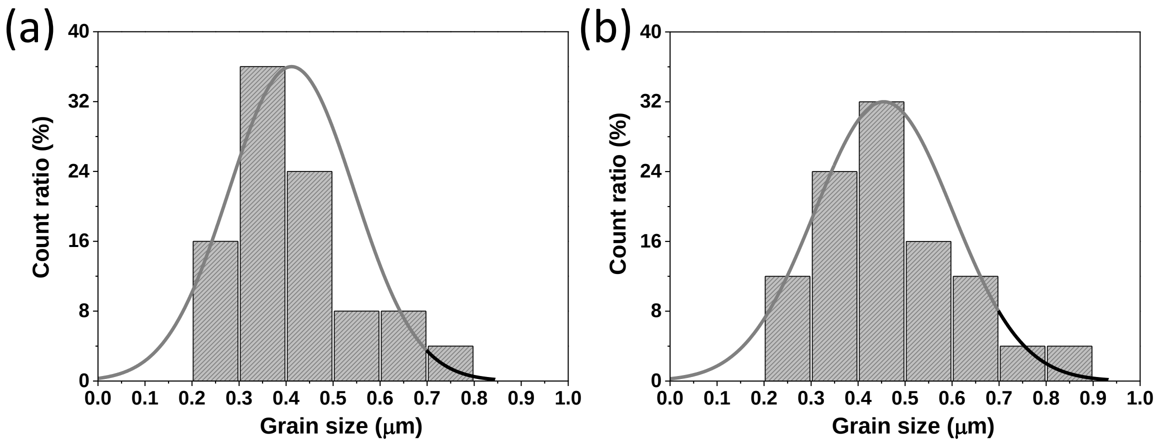

Figure S4. The crystal grain sizes based on SEM images of the perovskite thin films modified without and with CsBr, respetively.

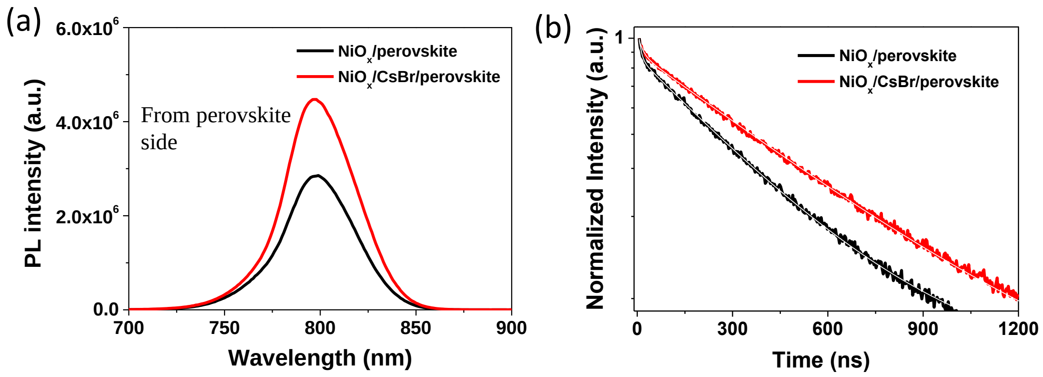

Figure S5. Steady-state PL (a) and time-resolved PL (b) spectra of perovskite films deposited on the pristine NiO_x and CsBr modified NiO_x thin films excited from air side. The average lifetimes are 424 ns for NiO_x/perovskite film and 618 ns for NiO_x/CsBr/perovskite film, respectively.

Table R1. Atomic percentage of the related elements in the thin film structure of (ITO/NiO_x/CsBr) and (ITO/NiO_x/CsBr/Perovskite (washed by DMF)) measured by EDX.

	Cs (%)	Pb (%)	I (%)	Br (%)
NiO_x/CsBr	1.54	-0.01	0.03	1.66
NiO_x/CsBr/perovskite (DMF washed)	1.39	0.02	-0.08	1.46

Table S2. Hysteresis behaviors of PSCs prepared on an HTL consisting of pristine NiO_x and NiO_x/CsBr. The hysteresis index (HI) is defined as ($PCE_{reverse}$-$PCE_{forward}$)/$PCE_{reverse}$.

Conditions		J_{sc} (mA/cm2)	V_{oc} (V)	FF	PCE (%)	HI
NiO_x	Reverse	22.58	1.08	0.73	17.89	0.45%
	Forward	22.88	1.07	0.73	17.81	
NiO_x/CsBr	Reverse	23.12	1.09	0.75	18.98	0.42%
	Forward	23.42	1.08	0.75	18.90	
